# Effects of vitamin D supplementation on the regulation of blood lipid levels in prediabetic subjects: A meta-analysis

**DOI:** 10.3389/fnut.2023.983515

**Published:** 2023-03-09

**Authors:** Yixue Yang, Shoumeng Yan, Nan Yao, Yinpei Guo, Han Wang, Mengzi Sun, Wenyu Hu, Xiaotong Li, Ling Wang, Bo Li

**Affiliations:** ^1^Department of Epidemiology and Biostatistics, School of Public Health, Jilin University, Changchun, China; ^2^School of Nursing, Jilin University, Changchun, Jilin, China

**Keywords:** vitamin D, meta-analysis, prediabetes, cholesterol, LDL cholesterol, HDL cholesterol, triglycerides

## Abstract

This meta-analysis aimed to systematically investigate whether vitamin D supplementation reduces blood lipid—total cholesterol (TC), LDL cholesterol (LDL-C), HDL cholesterol (HDL-C), and triglyceride (TG)—levels in prediabetic individuals. Pubmed, Web of Science, Cochrane Library, Embase, CNKI, and WANFANG databases were searched for studies published before 13 February 2022 (including 13 February 2022). Five articles were included. The results showed that vitamin D intervention led to a significant reduction in TG compared with control or placebo treatment (−0.42 [−0.59, −0.25], *P* < 0.001). Subgroup analyses showed that this effect was particularly significant among the studies that included obese subjects (−0.46 [−0.65, −0.28], *P* < 0.001), the studies that also included men (not only women) (−0.56 [−0.78, −0.34], *P* < 0.001), and the studies with intervention durations longer than 1 year (−0.46 [−0.65, −0.28], *P* < 0.001). Both relatively low doses of 2,857 IU/day (−0.65 [−0.92, −0.38], *P* < 0.001) and relatively high doses of 8,571 IU/day (−0.28 [−0.54, −0.02] *P* = 0.04) of vitamin D supplementation reduced TG levels, and the effect was observed both in Northern Europe (−0.65 [−0.92, −0.38], *P* < 0.001) and Asian (−0.25 [−0.48, −0.03], *P* = 0.03) country subgroups. No significant effects on TC, HDL-C, and LDL-C were shown. In conclusion, vitamin D supplementation might beneficially affect TG levels in individuals with prediabetes. Particularly longer durations of treatment, more than 1 year, with doses that correct vitamin deficiency/insufficiency, can have a beneficial effect. This meta-analysis was registered at www.crd.york.ac.uk/prospero (CRD42020160780).

## 1. Introduction

The global burden of diabetes mellitus cannot be ignored. In the last several decades, the prevalence of diabetes mellitus has extensively increased worldwide (1). It has been estimated that in 2011, there were approximately 366 million patients with diabetes and that the number is expected to reach 552 million by 2030 (2). Additionally, diabetes is associated with a high risk of cardiovascular diseases, mortality, and high economic costs related to the treatment and associated working disability (3, 4).

Diabetes can be preceded by prediabetes, and timely intervention during the prediabetic state is important for preventing the progression of diabetes (1, 5, 6). Prediabetes is defined as a state with a blood glucose level beyond the normal value but not reaching the diagnostic criteria for diabetes, including impaired fasting glucose (IFG, defined as fasting plasma glucose of 6.1–6.9 mmol/L or 5.6–6.9 mmol/L), impaired glucose tolerance (IGT, defined as 2h OGTT plasma glucose of 7.8–11.1 mmol/L), or glycated hemoglobin A1c (HbA1c) levels between 39 and 47 mmol/mol (7). Dyslipidemia is an important characteristic of both prediabetes and diabetes and may aggravate diabetic complications (8–11).

Vitamin D deficiency is another growing health concern in many parts of the world, affecting more than 50% of the general population worldwide (12). At the same time, it has been observed that people with lower 25(OH)D levels tend to have higher blood glucose (9), insulin resistance (10), and a higher risk of type 2 diabetes mellitus (T2DM) (11). In addition, some studies have shown that vitamin D supplementation may ameliorate dyslipidemia in subjects with T2DM (13, 14). Potential mechanisms included reduced intestinal cholesterol absorption, decreased low-density lipoprotein deposition in macrophages and foam cell formation, increased lipoprotein lipase gene expression in muscles and adipose tissue, etc. (15–17). However, there is still controversy over whether vitamin D supplementation can improve lipid levels in subjects with prediabetes since such studies are rare and more equivocal.

Therefore, we performed a meta-analysis evaluating the effect of vitamin D supplementation on blood lipid levels in subjects with prediabetes.

## 2. Materials and methods

### 2.1. Data sources and searches

**Figure 1:**
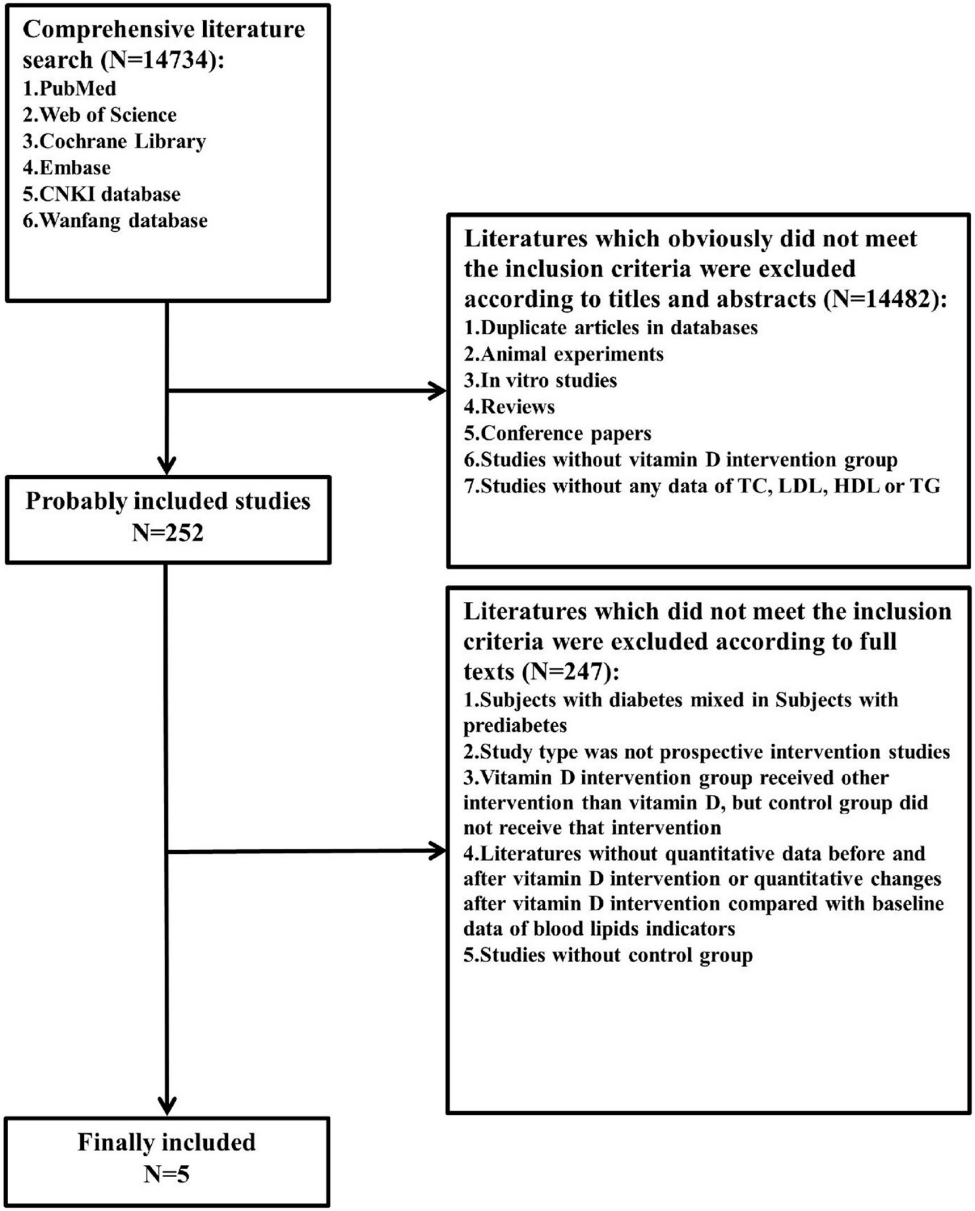
Flow diagram of the literature search and selection. TC, total cholesterol; LDL-C, low-density lipoprotein cholesterol; HDL-C, high-density lipoprotein cholesterol; TG, triglyceride.

**Figure 2:**
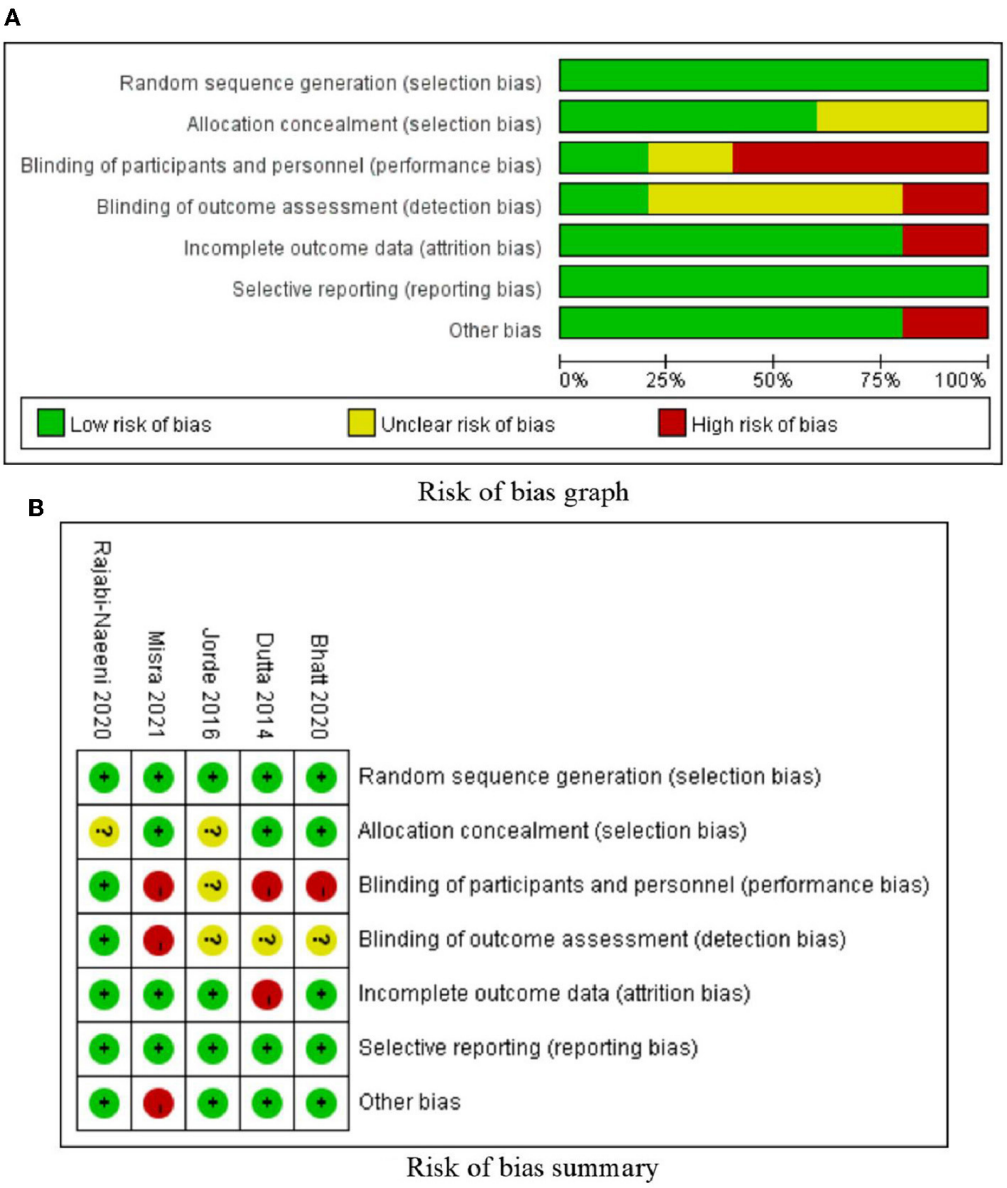
Risk of bias.

We comprehensively searched the PubMed, Web of Science, Cochrane Library, Embase, CNKI, and WANFANG databases for all studies with human subjects in any language published before 13 February 2022 (including 13 February 2022) (Figure 1). We explored changes in serum total cholesterol (TC), low-density lipoprotein cholesterol (LDL-C), high-density lipoprotein cholesterol (HDL-C), and triglyceride (TG) levels before and after intervention with vitamin D supplementation in comparison with changes in blood lipids on a control treatment without vitamin D supplementation. The control treatment was defined as no supplementation, placebo supplementation, or another treatment without vitamin D supplementation that was also present in the vitamin D supplementation group (e.g., lifestyle intervention, calcium carbonate supplementation, and omega-3 fatty acid supplementation). Two investigators independently reviewed the literature, discussed the inconsistencies, and worked independently during the selection process, data collection process, and study risk of bias assessment (Figure 2).

### 2.2. Inclusion and exclusion criteria

The included studies met the following criteria. Subjects should meet the diagnostic criteria for prediabetes (International Diabetes Federation (IDF)/World Health Organization (WHO) from 2006: fasting plasma glucose value in the range of 6.1–6.9 mmol/L or 110–125 mg/dl, or 2h oral glucose tolerance test (OGTT) plasma glucose value in the range of 7.8–11.0 mmol/L or 140–200 mg/dl (18) and American Diabetes Association (ADA) from 2004: fasting plasma glucose value in the range of 5.6–6.9 mmol/L or 100–125 mg/dl, or 2h OGTT plasma glucose value in the range of 7.8–11.0 mmol/L or 140–200 mg/dl; or HbA1c in the range of 39–47 mmol/mol or 5.7–6.4% (19). We restricted included studies to prospective intervention studies; studies included at least one vitamin D intervention group and one control group receiving no vitamin D supplementation, with the only difference between the intervention group and control group being vitamin D intervention; studies included at least one of the blood lipid indicators (TC, LDL-C, HDL-C, or TG); studies provided quantitative data before and after vitamin D intervention or quantitative changes after vitamin D intervention compared with baseline data of blood lipid indicators.

Duplicate articles in databases, studies that did not meet the above inclusion criteria, animal experiments, *in vitro* studies, reviews, and conference papers were excluded.

### 2.3. Data extraction

We read all included articles and then abstracted the following data: primary authors, nationality, and publication year; average age, gender, BMI, region, and the number of participants in each group; vitamin D supplement dose and time; criteria for prediabetes definition; TC, LDL-C, HDL-C, and TG alterations in intervention groups and control groups. If the included original article had more than one intervention group or control group, we chose the most suitable group for further analysis.

### 2.4. Quality assessment

Two investigators independently assessed the risk of bias using RevMan 5.3, including selection bias, performance bias, detection bias, attrition bias, reporting bias, and other bias, which were classified into three levels as high, low, or unclear, along with discussion and negotiation with respect to inconsistency. The judgment standards were established from Cochrane Handbook for Systematic Reviews of Interventions (20).

### 2.5. Statistical analysis

In this study, the RevMan 5.3 and Stata 12.0 software were used for statistical analysis. The average differences between the intervention group and the control group were calculated by the average changes in blood TC, LDL-C, HDL-C, and TG levels compared to baseline values (mean ± SD) (SD: standard deviation). When the original studies did not provide changes in SD, the formula in the Cochrane handbook was used to calculate (20).


$$\begin{array}{l} \text{S}{\text{D}}_{\text{change}}=\sqrt{\text{S}{\text{D}}_{\text{baseline}}^{2}+\text{S}{\text{D}}_{\text{end}}^{2}-\left(2\times \text{R}\times \text{S}{\text{D}}_{\text{baseline}}\times \text{S}{\text{D}}_{\text{end}}\right)} \end{array}$$

The correlation coefficient R of the equation was estimated using the baseline value, endpoint, and change values of blood lipids from other studies with vitamin D supplementation. Finally, the estimated R-value of this study was 0.84. The 95% confidence interval (CI), interquartile range (IQR), and 5th and 95th percentiles could also be transformed into SD (20) (1–0.95/2 = 0.025, then the value x was found using the formula = tinv(1–0.95, N-1) in Excel, where N means the population of this group).


$$\begin{array}{l} \text{SD}=\left[\sqrt{\text{N}}\times \left({95\text{\%CI}}_{\text{Upper limit}}-{95\text{\%CI}}_{\text{Lower limit}}\right)\right]÷2\text{x}\\ \text{SD}=\text{IQR}÷1.35=\left(95\text{th percentiles}-5\text{th percentiles}\right)÷3.29 \end{array}$$

The mean and SD of serum TC, LDL-C, HDL-C, and TG concentrations changes in the intervention group and the control group were compared by standardized mean difference (SMD). Cochran's Q-statistics and I^2^-statistics were used to evaluate the statistical heterogeneity in the meta-analysis. In a meta-analysis, the random effect model (REM) was used when data were heterogeneous, and the fixed effect model (FEM) was used when data were not heterogeneous (20), but model-using in subgroup analyses of TC, LDL-C, HDL-C, and TG remained consistent with the total meta-analysis of TC, LDL-C, HDL-C, and TG separately. In this study, SMD and 95% CI of TC and HDL-C changes were measured by REM; SMD and 95% CI of LDL-C and TG changes were measured by FEM; and data were compared between the vitamin D group and the control group.

In the Q-test, a *p*-value of <0.05 was indicative of heterogeneity, and the I^2^-value was used to evaluate the degree of heterogeneity. Influence analysis and Egger's test were performed using the Stata software to determine the stability and possible sources of heterogeneity. Combining the opinions of two investigators, the RevMan software was used for risk assessment. In addition, subgroup analysis was conducted according to BMI [overweight defined as 23–24.9, obesity as 25 or over 25 in Indian studies (21); overweight defined as 25–29.9, obesity as 30 or over 30 in other studies (22)], region (Northern Europe and Asia), vitamin D supplement dose (relatively low dose, relatively medium dose, and relatively high dose; according to included studies, we have found that doses of included studies were 2,857 IU/day, 3,571 IU/day, and 8,571 IU/day, therefore, we defined 2,857 IU/day as relatively low dose, 3,571 IU/day as relatively medium dose, and 8,571 IU/day as a relatively high dose in this meta-analysis), intervention time [short term defined as <365 days and long term defined as ≥365 days according to some articles (6, 23–29)], sex (only female or and male), and criteria for prediabetes definition (according to IDF/WHO or ADA criteria).

## 3. Results

### 3.1. Literature search

A total of 14,734 citations were retrieved, and only 5 papers fully met the inclusion criteria (5, 21, 23, 30, 31). These five articles included a sample of 601 subjects, with 311 in the vitamin D intervention group and 290 in the control group. Basic characteristics and details are shown in Table 1. The duration of treatment in the included studies ranged ≥56 days, and the dose of vitamin D ranged from 200 to 8,571 IU/day. In two studies, the control treatment involved only a placebo (23, 30); in the other two studies, placebo plus calcium carbonate supplementation (21, 31); and in one study, placebo plus calcium carbonate supplementation and lifestyle intervention (5) (note: also in the vitamin D intervention group, the same control treatments were applied).

Table 1Basic information of included studies.

**Table:** 

**References**	**Region**	**Intervention Dose (IU/D)**	**Intervention Duration (Day)**	**Study participants Total (intervention /control)**	**Sex[Female** ***N*** **(%)/Male** ***N*** **(%)]**		**Baseline BMI (kg/m** ^**2**^ **)**		**Baseline age**	
					**Intervention**	**Control**	**Intervention**	**Control**	**Intervention**	**Control**
**(A)**										
Bhatt et al. (21)	India	8571 or 200^a^	546	121 (61/60)	61 (100.0)/0 (0.0)	60 (100.0)/0 (0.0)	31.10 ± 6.20	28.80 ± 3.90	20-60	20-60
Dutta et al. (5)	India	8571^b^	56	104 (55/49)	33 (60.0)/22 (40.0)	26 (53.1)/23 (46.9)	26.32 ± 4.52	26.83 ± 4.63	48.37 ± 10.47	47.40 ± 11.51
		2000^b^	≥309							
Jorde et al. (23)	Norway	2857	365-1825	227 (116/111)	43 (37.1)/73 (62.9)	39 (35.1)/72 (64.9)	30.10 ± 4.10	29.80 ± 4.40	62.30 ± 8.10	61.90 ± 9.20
Rajabi-Naeeni et al. (30)	Iran	3571	56	84 (42/42)	42 (100.0)/0 (0.0)	42 (100.0)/0 (0.0)	27.01 ± 2.91	27.28 ± 2.74	39.92 ± 6.04	41.85 ± 7.48
Misra et al. (31)	India	8571 or 200^a^	720	65 (37/28)	37 (100.0)/0 (0.0)	28 (100.0)/0 (0.0)	–	–	48.10 ± 6.70	46.10 ± 8.10

BMI data are shown as mean ± SD; age data are shown as mean ± SD or age range; original doses were converted into doses per day and were rounded if they were not integers; 1 month was converted into 30 days. –Data were unavailable or could not be calculated.

a Intervention dose: gave 60 000 IU/week for the first 8 weeks, adjusted doses every 24 weeks according to blood 25(OH)D levels, gave 60,000 IU/week for 8 weeks to subjects with vitamin D deficient, gave 200 IU/day to subjects with normal blood 25(OH)D level.

b Intervention dose: 60,000 U/W for the first 8 weeks, then 60,000 U/M, subjects were followed up for at least 12 months. BMI, body mass index; SD, standard deviation.

**Table:** 

**References**	**TC change (mmol/L)**		**LDL-C change (mmol/L)**		**HDL-C change (mmol/L)**		**TG change (mmol/L)**	
	**Intervention**	**Control**	**Intervention**	**Control**	**Intervention**	**Control**	**Intervention**	**Control**
**(B)**								
Bhatt et al. (21)	−0.18 ± 0.60	0.26 ± 0.61	0.12 ± 0.49	0.07 ± 0.44	0.08 ± 0.16	0.03 ± 0.17	−0.10 ± 0.48	−0.01 ± 0.37
Dutta et al. (5)	–	–	−0.25 ± 0.46	−0.18 ± 0.36	−0.12 ± 0.15	−0.03 ± 0.16	−0.06 ± 0.40	0.09 ± 0.41
Jorde et al. (23)	−0.41 ± 0.59	−0.50 ± 0.59	−0.14 ± 0.52	−0.18 ± 0.52	0.09 ± 0.21	0.04 ± 0.21	−0.20 ± 0.43	0.10 ± 0.49
Rajabi-Naeeni et al. (30)	−0.27 ± 0.53	−0.11 ± 0.49	−0.27 ± 0.46	−0.11 ± 0.44	0.01 ± 0.16	−0.01 ± 0.13	0.04 ± 0.49	0.12 ± 0.39
Misra et al. (31)	−0.85 ± 0.66	−0.86 ± 0.87	–	–	–	–	–	–

Data are shown as mean ± SD. – Data were unavailable or could not be calculated. HDL-C, high-density lipoprotein cholesterol; TG, triglyceride; TC, total cholesterol; LDL-C, low-density lipoprotein cholesterol; SD, standard deviation.

As shown in Table 1, four studies were conducted in Asia and one in Northern Europe; one study excluded obese subjects (i.e., only normal weight and overweight subjects were included), three studies included obese subjects, and one did not provide BMI information. The experimental group in one study was supplemented with relatively low-dose vitamin D, one was supplemented with relatively medium-dose vitamin D, and three were supplemented with relatively high-dose vitamin D; one study carried out short-term interventions, and four carried out long-term interventions; three studies only had female subjects, and two had both male and female subjects; Four studies used ADA criteria for prediabetes; one used IDF/WHO criteria.

### 3.2. Risk of bias assessment

The risk of bias is shown in Figures 2A, B. Systematically speaking of this meta-analysis, the risks of random sequence generation bias and reporting bias in the included studies were very low; the risks of allocation concealment bias, attrition bias, and other biases were low; the risk of detection bias was relatively low; and the risk of performance bias was relatively high.

### 3.3. Meta-analysis

**Figure 3:**
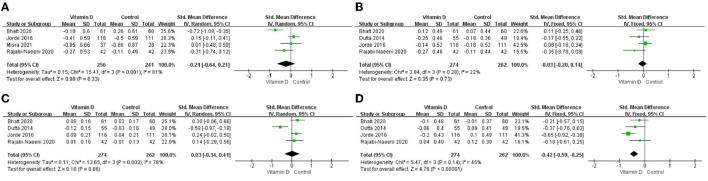
Forest map of lipid changes between two groups. **(A)** TC, **(B)** LDL–C, **(C)** HDL–C, and **(D)** TG. TC, total cholesterol; LDL-C, low-density lipoprotein cholesterol; HDL-C, high-density lipoprotein cholesterol; TG, triglyceride.

The differences in TG change (*P* < 0.001; Figure 3D, Table 2) between the intervention group and the control group were statistically significant. Compared to the control group, TG in the blood in the intervention group decreased more after vitamin D supplementation. But there were no significant differences in blood TC change (*P* = 0.33; Figure 3A, Table 2), blood LDL-C change (*P* = 0.73; Figure 3B, Table 2), or blood HDL-C change (*P* = 0.86; Figure 3C, Table 2) between the intervention group and the control group.

**Table 2:** Results of meta-analysis in five included articles.

**Index**	**Number of Studies**	**SMD [95%CI]**	***I* ^2^**
TC	4	−0.21 [−0.64,0.21]	**81%** ^**‡**^
LDL-C	4	−0.03[−0.20, 0.14]	22%
HDL-C	4	0.03 [−0.34, 0.41]	**78%** ^**‡**^
TG	4	**−0.42 [−0.59**, **−0.25]**^**†**^	45%

† P < 0.05 of the test for overall effect;

‡ P < 0.05 of the test for heterogeneity. SMD, standardized mean difference; CI, confidence interval.

### 3.4. Subgroup analysis results

**Figure 4:**
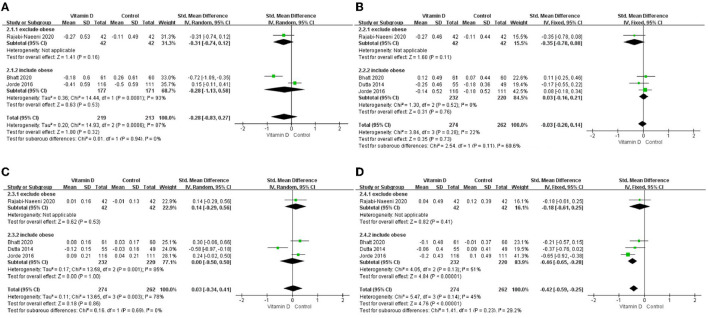
Subgroup analysis by BMI. **(A)** TC, **(B)** LDL–C, **(C)** HDL–C, and **(D)** TG. BMI, body mass index; TC, total cholesterol; LDL-C, low-density lipoprotein cholesterol; HDL-C, high-density lipoprotein cholesterol; TG, triglyceride.

**Figure 5:**
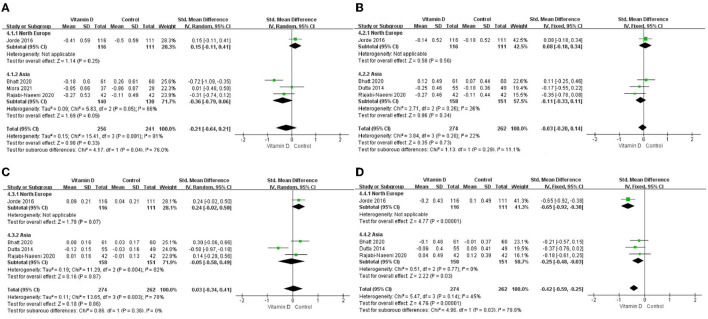
Subgroup analysis by region. **(A)** TC, **(B)** LDL–C, **(C)** HDL–C, and **(D)** TG. TG. TC, total cholesterol; LDL-C, low-density lipoprotein cholesterol; HDL-C, high-density lipoprotein cholesterol; TG, triglyceride.

**Figure 6:**
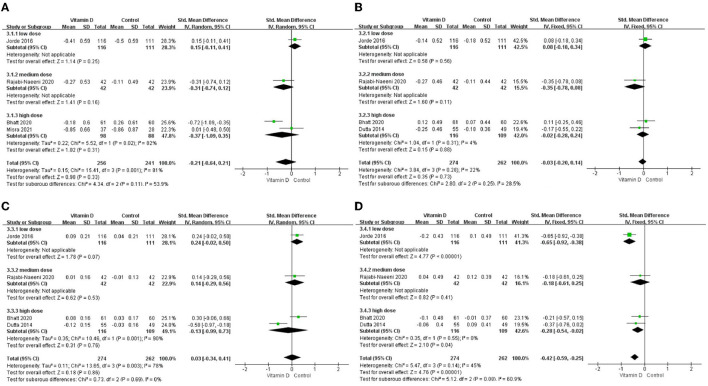
Subgroup analysis by intervention dose. **(A)** TC, **(B)** LDL-C, **(C)** HDL-C, and **(D)** TG. TC, total cholesterol; LDL-C, low-density lipoprotein cholesterol; HDL-C, high-density lipoprotein cholesterol; TG, triglyceride.

**Figure 7:**
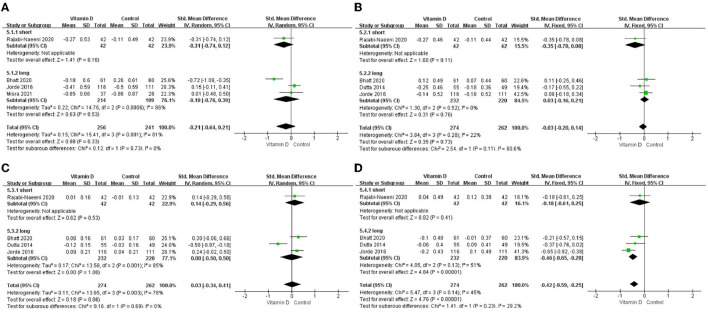
Subgroup analysis by intervention duration. **(A)** TC, **(B)** LDL-C, **(C)** HDL-C, and **(D)** TG. TC, total cholesterol; LDL-C, low-density lipoprotein cholesterol; HDL-C, high-density lipoprotein cholesterol; TG, triglyceride.

**Figure 8:**
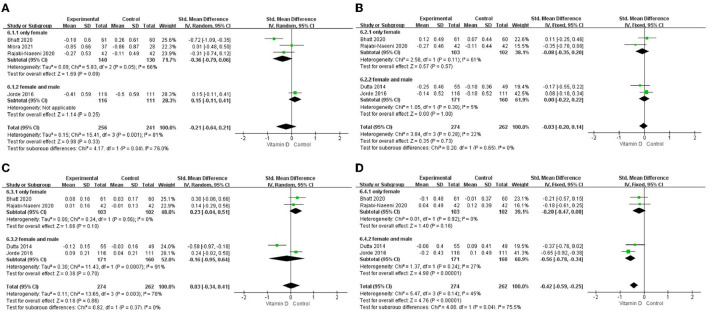
Subgroup analysis by sex. **(A)** TC, **(B)** LDL-C, **(C)**, HDL-C, and **(D)** TG. TC, total cholesterol; LDL-C, low-density lipoprotein cholesterol; HDL-C, high-density lipoprotein cholesterol; TG, triglyceride.

**Figure 9:**
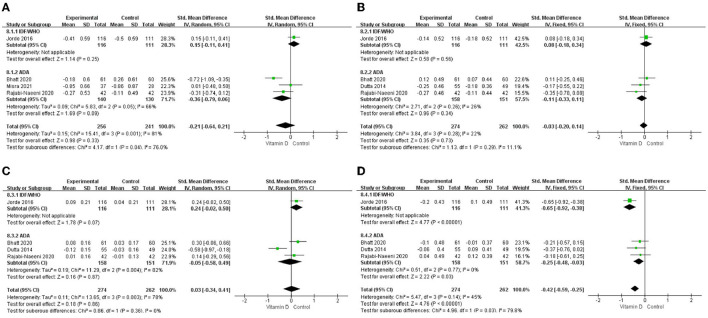
Subgroup analysis by criteria for prediabetes definition. **(A)** TC, **(B)** LDL-C, **(C)** HDL-C, and **(D)** TG. TC, total cholesterol; LDL-C, low-density lipoprotein cholesterol; HDL-C, high-density lipoprotein cholesterol; TG, triglyceride; IDF, International Diabetes Federation; ADA, American Diabetes Association; WHO, World Health Organization; Criteria of IDF/ WHO from 2006: fasting plasma glucose value in the range of 6.1–6.9 mmol/L or 110–125 mg/dl, or 2h oral glucose tolerance test (OGTT) plasma glucose value in the range of 7.8–11.0 mmol/L or 140–200 mg/dl; Criteria of ADA from 2004: fasting plasma glucose value in the range of 5.6–6.9 mmol/L or 100–125 mg/dl, or 2 h OGTT plasma glucose value in the range of 7.8–11.0 mmol/L or 140–200 mg/dl; or HbA1c in the range of 39–47 mmol/mol or 5.7–6.4%.

Only in the subgroup of studies that included obese subjects (not only normal weight and overweight), vitamin D supplementation led to more reductions in TG levels compared to the control treatments (*P* < 0.001; Figure 4). The effect of vitamin D supplementation on TG levels was observed both in the subgroup of Asian countries (*P* = 0.03; Figure 5) and the subgroup of Northern European counties (*P* < 0.001; Figure 5), and both in the relatively high-dose subgroup (*P* = 0.04; Figure 6) and in the relatively low-dose subgroup (*P* < 0.001; Figure 6). Only in the long-term intervention subgroup (more than 1 year of vitamin D supplementation) (*P* < 0.001; Figure 7) and only in the subgroup with both female and male subjects included (not only females) (*P* < 0.001; Figure 8), the effect of vitamin D supplementation on TG levels was observed. Both in the IDF/WHO subgroup (*P* < 0.001; Figure 9) and the ADA subgroup (*P* = 0.03; Figure 9), vitamin D supplementation led to more reductions in TG levels compared to the control treatments. It was noteworthy to emphasize that studies with obese subjects included were all long-term interventions (more than 1 year of vitamin D supplementation), while the only study (30) with non-obese subjects included was at the same time a short-term intervention (only 56 days), and the vitamin D levels in that study were not corrected at the end of the study (they remained insufficient). Additionally, this short-term intervention study was the only study with a relatively medium dose included, where the effect was not observed (*P* = 0.41; Figures 4, 6, 7).

The effects on TC in obese, relatively high-dose, and long-time subgroups and on HDL-C in obese, relatively high-dose, Asian, long-time, female and male, and ADA subgroups showed heterogeneity (Table 3).

Table 3Results of subgroup meta-analysis.

**Table:** 

	**Index**	**BMI Subgroup**		**Dose Subgroup**			**Region Subgroup**	
		**Exclude Obese**	**Include Obese**	**Low Dose**	**Medium Dose**	**High Dose**	**Northern Europe**	**Asia**
**(A)**								
Number of Studies	TC	1	2	1	1	2	1	3
	LDL-C	1	3	1	1	2	1	3
	HDL-C	1	3	1	1	2	1	3
	TG	1	3	1	1	2	1	3
SMD [95%CI]	TC	−0.31 [−0.74, 0.12]	−0.28 [−1.13, 0.58]	0.15 [−0.11, 0.41]	−0.31 [−0.74, 0.12]	−0.37 [−1.09, 0.35]	0.15 [−0.11, 0.41]	−0.36 [−0.79, 0.06]
	LDL–C	−0.35 [−0.78, 0.08]	0.03 [−0.16, 0.21]	0.08 [−0.18, 0.34]	−0.35 [−0.78, 0.08]	−0.02 [−0.28, 0.24]	0.08 [−0.18, 0.34]	−0.11 [−0.33, 0.11]
	HDL–C	0.14 [−0.29, 0.56]	0.00 [−0.50, 0.50]	0.24 [−0.02, 0.50]	0.14 [−0.29, 0.56]	−0.13 [−0.99, 0.73]	0.24 [−0.02, 0.50]	−0.05 [−0.58, 0.49]
	TG	−0.18 [−0.61, 0.25]	**−0.46 [−0.65**, **−0.28]**^**†**^	**−0.65 [−0.92**, **−0.38]**^**†**^	−0.18 [−0.61, 0.25]	**−0.28 [−0.54**, **−0.02]**^**†**^	**−0.65 [−0.92**, **−0.38]**^**†**^	**−0.25 [−0.48**, **−0.03]**^**†**^
I^2^	TC	–	**93%** ^**‡**^	–	–	**82%** ^**‡**^	–	66%
	LDL-C	–	0%	–	–	4%	–	26%
	HDL-C	–	**85%** ^**‡**^	–	–	**90%** ^**‡**^	–	**82%** ^**‡**^
	TG	–	51%	–	–	0%	–	0%

**Table:** 

	**Index**	**Duration subgroup**		**Sex subgroup**		**Prediabetes criteria subgroup**	
		**Short**	**Long**	**Only female**	**Female and male**	**IDF/WHO**	**ADA**
**(B)**							
Number of Studies	TC	1	3	3	1	1	3
	LDL-C	1	3	2	2	1	3
	HDL-C	1	3	2	2	1	3
	TG	1	3	2	2	1	3
SMD [95%CI]	TC	−0.31 [−0.74, 0.12]	−0.18 [−0.76, 0.39]	−0.36 [−0.79, 0.06]	0.15 [−0.11, 0.41]	0.15 [−0.11, 0.41]	−0.36 [−0.79, 0.06]
	LDL-C	−0.35 [−0.78, 0.08]	0.03 [−0.16, 0.21]	−0.08 [−0.35, 0.20]	0.00 [−0.22, 0.22]	0.08 [−0.18, 0.34]	−0.11 [−0.33, 0.11]
	HDL-C	0.14 [−0.29, 0.56]	0.00 [−0.50, 0.50]	0.23 [−0.04, 0.51]	−0.16 [−0.95, 0.64]	0.24 [−0.02, 0.50]	−0.05 [−0.58, 0.49]
	TG	−0.18 [−0.61, 0.25]	**−0.46 [−0.65**, **−0.28]**^**†**^	−0.20 [−0.47, 0.08]	**−0.56 [−0.78**, **−0.34]**^**†**^	**−0.65 [−0.92**, **−0.38]**^**†**^	**−0.25 [−0.48**, **−0.03]**^**†**^
I^2^	TC	–	**86%** ^**‡**^	66%	–	–	66%
	LDL-C	–	0%	61%	5%	–	26%
	HDL-C	–	**85%** ^**‡**^	0%	**91%** ^**‡**^	–	**82%** ^**‡**^
	TG	–	51%	0%	27%	–	0%

† P < 0.05 of the test for overall effect;

‡ P < 0.05 of the test for heterogeneity. BMI, body mass index; SMD, standardized mean difference; CI, confidence interval. BMI definition: in Indian studies, overweight = 23–24.9, obesity≥25; in other studies, overweight = 25–29.9, obesity≥30. Dose definition: relatively low-dose = 2,857 IU/day; relatively medium dose = 3,571 IU/day; relatively high dose = 8,571 IU/day. Duration definition: short-term <365 days; long-term≥365 days. Bold values: P < 0.05.

### 3.5. Influence analysis and Egger's test

**Figure 10:**
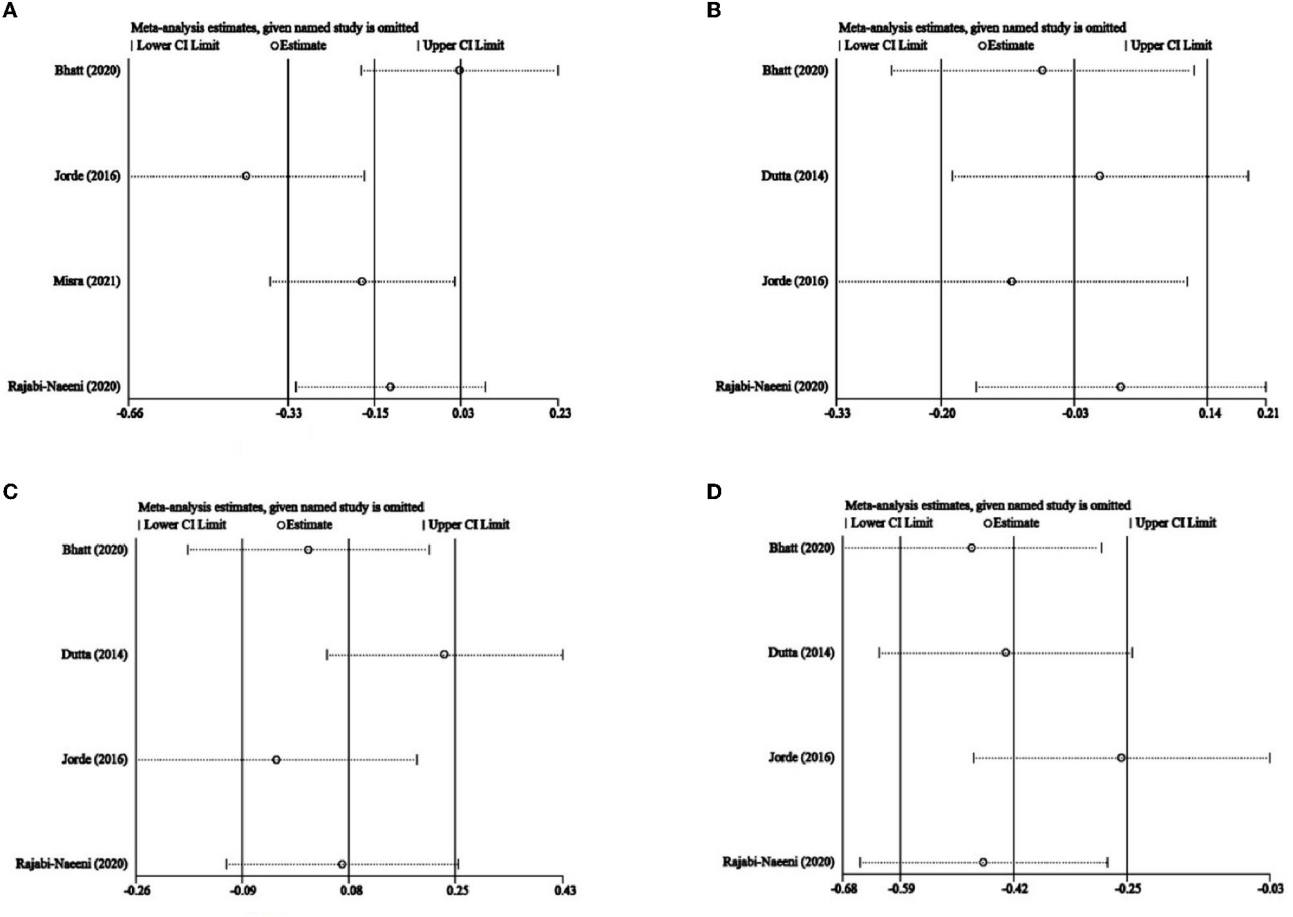
Influence analysis. **(A)** TC, **(B)** LDL-C, **(C)**, and HDL-C, and **(D)** TG. TC, total cholesterol; LDL-C, low-density lipoprotein cholesterol; HDL-C, high-density lipoprotein cholesterol; TG, triglyceride.

The impact of every single article on heterogeneity was observed in Figure 10. The study by Jorde et al. (23) seemed different from the others in this meta-analysis of TC. In addition, according to Egger's test, there was no significant publication bias in any of the included articles for TC (*P* = 0.540), LDL-C (*P* = 0.213), HDL-C (*P* = 0.529), or TG (*P* = 0.096).

## 4. Discussion

The results of our meta-analysis showed that vitamin D supplementation could decrease circulating TG levels in subjects with prediabetes, especially in certain situations, but failed to confirm the effects on TC, HDL-C, and LDL-C levels.

Many studies have shown that low serum 25(OH)D concentration was associated with adverse lipid status (32), and some studies indicated that vitamin D supplementation could improve serum TC, TG, and LDL-C levels also in patients with T2DM (13, 14) and in subjects with metabolic syndrome (33).

The effect of vitamin D supplementation on TG levels can be mediated through (1) increased calcium levels; (2) suppression of parathyroid hormone (PTH) secretion; (3) inhibition of lipolysis; (4) suppression of inflammation; (5) suppression of renin-angiotensin-aldosterone system (RAAS) activity; (6) its interaction with glucocorticoids and sex hormones; (7) upregulation of adiponectin; (8) improvement in insulin resistance and insulin levels; (9) its direct inhibition of the expression of nuclear factor sterol regulatory element-binding protein 1c (SREBP1c) involved in hepatic TG synthesis; (10) increased TG clearance by upregulation of lipoprotein lipase (LPL), neutral sphingomyelinases, PPARγ, and adipocyte-binding protein 2 (AP2); or by (11) upregulation of mitochondrial oxidation (34–41).

Since adipose tissue can sequestrate and metabolize vitamin D and consequently lower its circulating and bioavailable levels for other metabolically active tissues involved in lipid metabolism (including muscle, liver, and pancreas) (42–45), we conducted an additional stratified analysis according to BMI categories. Our stratified analysis has shown that, particularly in the studies (5, 21, 23) that included obese subjects with prediabetes (not only normal weight and overweight subjects), the effect on TG levels was more marked compared with the study that excluded obese subjects (30). This result might be affected by the fact that in some of the studies that included obese subjects, men were also included (not only women). Our sex-subgroup analysis showed that the effect on TG was more marked in the mixed-sex studies (5, 23) than in the studies that included only women (21, 30) where the effect was not significant. Additionally, the durations of interventions in the studies (5, 21, 23) that also included obese subjects were over 1 year, while the duration of the treatment in the study that excluded obese subjects (30) was 8 weeks. Those factors can be significant confounders, which need to be taken into consideration when making conclusions. Nevertheless, there might be a more direct association. For example, obese subjects can have much higher TG levels compared with non-obese subjects, and therefore the effect can be more observable, especially during prolonged treatment. An additional explanation could be that obese subjects are more prone to vitamin D deficiency, while improvements in insulin resistance and related metabolic features can be observed after its correction. However, the later explanation failed to be confirmed in this study. In the study by Rajabi-Naeeni et al. (30) (where the effect on TG was not shown), the included normal weight and overweight subjects were with vitamin D insufficient status, in the study by Bhatt et al. (21) (where the effect was also not shown) the included overweight and obese subjects were with vitamin D deficient status, while in the studies by Dutta et al. (5) and Jorde et al. (23) (the ones which have found the significant effect on TG), they were with vitamin D insufficient status and no BMI restrictions. Therefore, the effect was neither associated with the baseline vitamin D status nor the BMI status, which is in agreement with a recent pooled meta-analysis (46). However, it is important to say that in the study by Bhatt et al. (21), the BMI-cut off for obesity was set at a much lower level (>25 kg/m^2^) according to Indian references, whereas in the other studies, it was set at the BMI-cut off for overweight. Finally, the effect cannot be explained by the rationale that vitamin D supplementation could affect body weight since not enough evidence exists on the effect of vitamin D on body weight reduction (42, 43, 47, 48), and no significant reductions in BMI were shown in the analyzed studies by vitamin D supplementation in comparison with the control treatments (5, 21, 23). Therefore, the finding of the more pronounced effect on TG in the studies which also included obese subjects was probably confounded by the influence of duration of treatment and/or possible gender differences in the response to supplementation (49).

As there are huge ethical/regional differences in vitamin D levels and responses to supplementation (50–56), we also conducted a region-subgroup analysis. The results showed that the effect on TG was not region-specific and was observed in both region-subgroups (Asia and Northern Europe). However, since this meta-analysis only included studies from India (three studies), Iran (one study), and Norway (one study), the results probably cannot be extrapolated to other regions or ethnicity subgroups, and relative trials in more regions are needed.

We conducted a subgroup analysis of intervention dose and time according to some studies with the idea that insufficient vitamin D dose and intervention time might affect the research results (24). The results showed that in our relatively low-dose (2,857 IU/day) and relatively high-dose (8,571 IU/day) subgroups, vitamin D intervention provided significantly larger reductions in TG, but such an effect was not observed in the relatively medium-dose subgroup. However, the relatively medium-dose group only included one study by Rajabi-Naeeni et al. (30), which was at the same time the only study in the short-duration group. Changes in glucose tolerance and blood lipid levels are usually a slow and gradual process, and previous research suggests that interventions lasting only a few months may be a too short time frame to evaluate the benefits of vitamin D, implying that even 1 year is not enough for a long-term intervention (6, 24, 25, 33). In our analysis, significantly larger reductions in TG levels in the intervention group had been observed only in the long-duration subgroup but not in the short-duration subgroup. Additionally, the vitamin D levels in the short-duration subgroup of the study (30) did not change to achieve vitamin D sufficiency after only 8 weeks of treatment. This implied that the relatively medium-dose group in our analysis was probably affected by the short duration of the study by Rajabi-Naeeni et al. (30). This is in agreement with a recent meta-analysis in subjects with metabolic syndrome, where the effects on TG levels were not shown in studies lasting <1 year (33). More short-term interventions are needed for further verification. Additionally, lower vitamin D doses than 2,857 IU/day or higher doses than 8,571 IU/day need to be tested in the future.

We have also conducted subgroup analyses of the criteria for prediabetes. The results showed that the effect on TG was probably not affected by different criteria for prediabetes since it was observed in both criteria subgroups (IDF/WHO and ADA).

Our heterogeneity might come from studies with different study designs (different control interventions, doses, durations, different inclusion criteria, baseline vitamin D status and corrections achieved, BMI, sex, and ethnicity of the subjects included). On the one hand, the significant heterogeneity disappeared after region, sex, and criteria for prediabetes subgroup analysis of TC, showing that region, gender, or criteria for prediabetes could affect the heterogeneity of our meta-analysis of TC. On the other hand, Figure 10 shows that the included article of Jorde et al. (23) was quite different from the others in this meta-analysis of TC. In the present study, in the subgroups with a North European population and relatively low dose and IDF/WHO prediabetic inclusion criteria, only the study of Jorde et al. (23) was included while regarding sex subgroups, only this study had a higher percentage of male participants. This suggested that the heterogeneity of this meta-analysis might come from the region, intervention dose, criteria of prediabetes, and sex of participants in the article by Jorde et al. (23). Besides, although it was not observed in Figure 10, the article by Rajabi-Naeeni et al. (30) was exceptional as well, with a relatively medium dose, a short duration, and the exclusion of obese subjects. Therefore, significant heterogeneity might also come from BMI, intervention dose, and intervention duration of the article by Rajabi-Naeeni et al. (30).

To make our analysis an all-around study as far as possible, we restricted our selection to prospective intervention trials based on the Cochrane Handbook for Systematic Reviews of Interventions and relative references (20, 57). Limitations of this meta-analysis were that there were not many studies (especially with normal BMI, more other regions, lower vitamin D doses than 2,857 IU/day and higher doses than 8,571 IU/day, and a low intervention duration of vitamin D supplementation) that could be included in this meta-analysis. More studies are needed in the future. Several included studies did not state clearly if they collected blood lipid data when participants were fasting, and only few of the included studies controlled for the usage of lipid lowering medications in the study by Bhatt et al. (21), no information on lipid-lowering drugs usage was provided; in the study by Dutta et al. (5), participants using metformin, fever/active oral hypoglycaemic agents, oral contraceptive pills, steroids, and anti-epileptics were excluded; in the study by Jorde et al. (23), participants with use of statins were included, but there was no significant difference in use of statins between groups and those who changed their use of statins during the course of study, were excluded; in the study by Misra et al. (31), participants on medications within last 1 month which could potentially influence insulin secretion, insulin sensitivity, vitamin D, or calcium metabolism, including metformin, thiazolidinediones, steroids, and calcitonin were excluded; and in the study by Rajabi-Naeeni et al. (30), participants using herbal or chemical medications affecting lipids were excluded). Besides, there was significant heterogeneity in the studies included, related to different durations and doses of treatment, different BMI and gender of the subjects included, different regions and ethnic populations, different criteria for prediabetes, different control interventions, baseline vitamin D status, and corrections achieved.

However, despite all these limitations, our results may provide a basis for the implementation of regular assessment of vitamin D status among patients with prediabetes and consecutive supplementation in vitamin D deficient/insufficient patients to prevent an increase in blood lipids.

## 5. Conclusion

Vitamin D supplementation might beneficially affect TG levels in individuals with prediabetes. Particularly longer durations of treatment, more than 1 year, with doses that correct vitamin deficiency/insufficiency, can have a beneficial effect. Considering that there were not many studies that could be included in this meta-analysis, more studies are needed in the future.

## Author contributions

YY and SY designed the study and analyzed the data. YY drafted the first manuscript and conducted the visualization. NY, YG, HW, MS, WH, XL, and LW validated the results. YY, SY, and BL participated in amending the manuscript. All authors contributed to the article and approved the submitted version.
